# Review of Hepatitis E Virus in Rats: Evident Risk of Species *Orthohepevirus C* to Human Zoonotic Infection and Disease

**DOI:** 10.3390/v12101148

**Published:** 2020-10-09

**Authors:** Gábor Reuter, Ákos Boros, Péter Pankovics

**Affiliations:** Department of Medical Microbiology and Immunology, Medical School, University of Pécs, H-7624 Pécs, Hungary; borosakos@gmail.com (Á.B.); pankovics.peter@gmail.com (P.P.)

**Keywords:** hepatitis E virus, hepevirus, orthohepevirus, rodent, rat, zoonosis, reservoir, cross-species transmission, hepatitis, human

## Abstract

Hepatitis E virus (HEV) (family *Hepeviridae*) is one of the most common human pathogens, causing acute hepatitis and an increasingly recognized etiological agent in chronic hepatitis and extrahepatic manifestations. Recent studies reported that not only are the classical members of the species *Orthohepevirus A* (HEV-A) pathogenic to humans but a genetically highly divergent rat origin hepevirus (HEV-C1) in species *Orthohepevirus C* (HEV-C) is also able to cause zoonotic infection and symptomatic disease (hepatitis) in humans. This review summarizes the current knowledge of hepeviruses in rodents with special focus of rat origin HEV-C1. Cross-species transmission and genetic diversity of HEV-C1 and confirmation of HEV-C1 infections and symptomatic disease in humans re-opened the long-lasting and full of surprises story of HEV in human. This novel knowledge has a consequence to the epidemiology, clinical aspects, laboratory diagnosis, and prevention of HEV infection in humans.

## 1. Introduction

Hepatitis E virus (HEV) is one of the most common pathogens causing acute hepatitis in tropical and subtropical areas and an increasingly recognized pathogen in acute, chronic hepatitis and extrahepatic manifestations in developed countries [1]. In general, HEV has a positive-sense, single-stranded 6.6–7.3kb-long RNA genome containing three open reading frames (ORFs) [2]. The ORF1, ORF2, and ORF3 encode the viral non-structural, capsid, and a small immunoreactive proteins, respectively. Hepeviruses are members of the family *Hepeviridae* and are classified into two genera: *Orthohepevirus* and *Piscihepevirus* [2]. Genus *Orthohepevirus* can be divided into four species: *Orthohepevirus A–D*. Within the species *Orthohepevirus A*, at least 8 distinct genotypes (HEV-1 to HEV-8) and several sub-genotypes have been identified [2,3,4]. It should also be noted that there are several further unassigned hepeviruses in the family which predict future significant change in the taxonomy of the virus family. The known sources and transmission routes of HEV infections in human are diverse and—based on the present knowledge—influenced by the genetic variants of the virus. Historically, it is generally thought that HEV is transmitted by human-to-human in developing countries because of the low social and hygienic conditions and only travel-related imported cases occur in developed countries. The most important initial breakthrough was the first detection of HEV in animal species, in swine in 1997 [5], which confirmed, for the first time, the possibility of the animal-to-human transmission of HEV as a zoonosis.

## 2. HEV in Animals as a Source of Human Infections

Orthohepeviruses have been identified from human, mammalian and avian species. Now, it is clear that wide range of animals are reservoirs and hosts for hepeviruses in genus *Orthohepevirus*, and some of them are confirmed as a potential source for human infection, too. Hepeviruses in species *Orthohepevirus A* are able to spread as a zoonosis from mammals to humans. Until now, swine, wild boar, deer, rabbit, and camel are present on a list as confirmed sources of human HEV infections. In species *Orthohepevirus A*, genotypes HEV-1 and HEV-2 infect only humans [4], HEV-3 and HEV-4 infect humans and several animal species [4], HEV-5 and HEV-6 infect wild boars, HEV-7 infects dromedary camels and humans [6,7], and HEV-8 infects Bactrian camels and cynomolgus macaques [8,9]. HEV-1 and HEV-2 are the main known to obligate human pathogens in tropical and subtropical areas (in Asia, Africa, and Mexico), while HEV-3 is the leading genotype in endemic acute and chronic hepatitis in human in Europe and the USA. Among HEVs, HEV-3 is the most frequent animal-to-human zoonotic genotype transmitted via virus-contaminated food, livestock, or organ transplantation and blood products during transfusion [10,11]. HEV-4 is also zoonotic, able to spread from animals (mostly pigs) to humans, but, until now, is less often identified than HEV-3 in Asia and Europe [11]. There is only one reported case of potential transmission of HEV-7 from camel to human [7]. In addition, recent studies reported that not only are the members of the species *Orthohepevirus A* pathogenic to humans. Interestingly, genetically highly divergent (<52%) rat origin hepevirus (HEV-C1) in species *Orthohepevirus C* is also able to cause infection and symptomatic human disease (hepatitis) [12,13]. This knowledge opens up new, unexplored fields of human HEV infection related to the potential host species spectrum and human-infecting HEV variants. Table 1 summarizes the presently known host species and reservoirs of orthohepeviruses, including the confirmed animals which play a role in interspecies transmissions of orthohepeviruses from animals to humans (Table 1).

## 3. Frequency, Epidemiology, and Geographic Distribution of HEV-C in Rats

The first rodent-associated hepevirus was discovered from fecal and liver specimens from rats in Germany in 2010 (Table 2) [14,15] and has been classified into species *Orthohepevirus C* genotype HEV-C1. Since then, rat hepevirus (HEV-C1) has been detected in multiple wild rat species, including in wild Norway rats (*Rattus norvegicus*) in Germany [14,15] and another 15 countries (Table 2), Black rats (*Rattus rattus*) from USA [16] and Indonesia [17], Tanezumi rats (*Rattus tanezumi*) from Vietnam [18], and Bandicoot rats (*Bandicota indica*), *Rattus flavipectus*, and *Rattus rattoides losea* from China [19]. Rat HEV-C1 was detected in 12 European countries (Austria, Belgium, Czech Republic, Denmark, France, Germany, Greece, Hungary, Italy, Lithuania, Spain, and Switzerland), and in Norway and Black rats [20,21]. These studies suggest that HEV-C1 is geographically widespread among rats and present in a minimum of three continents (Asia, America, and Europe) (Table 2). No association with age, sex, or the *Rattus* species and the individual rat hepevirus (HEV-C1) infection status could be detected in Europe indicating a potential non-persistent infection [20]. In addition, interestingly, the infectious potential of rat hepeviruses are wider than we previously thought. According to a Chinese study, Asian musk shrew (*Suncus murinus*) seems to be also a reservoir of HEV-C1 (Table 2) because of its close/same environment with rat [22]. Interestingly, rat HEV-C1 was identified from Syrian brown bear in a zoo animal in Germany [23], most likely the result of a spillover infection from rats, based on the close phylogenetic relationship with the wild rat HEV-C1 described in Germany, too [15]. It should be noted that species *Orthohepevirus C* contains hepevirus in other small mammals and rodents than rat, too. HEV-C2 discovered in ferret [24] and mink [25] and recent reports described further unassigned, genetically novel rodent HEV-C strains from Chevrier’s field mouse (*Apodemus chevrieri*), Pere David’s vole (*Eothenomys melanogaster*) [26], related to the first identified vole-borne HEV-C hepevirus strain in this group from carnivorous bird species, kestrel and falcon possibly due to its diet [27], to Hairy-tailed bolo mouse (*Necromys lasiurus*) and Delicate vesper mouse (*Calomys tener*) [28]. On the other hand, it should be also noted that HEV-3 (*Orthohepevirus A*) was also detected from rat (Table 2), but finding of this strain in rat is controversial and may depend on the habitat sampled [20,29].

## 4. Genome Characterization and Sequence Analysis of Rodent Hepeviruses

**Figure 1:**
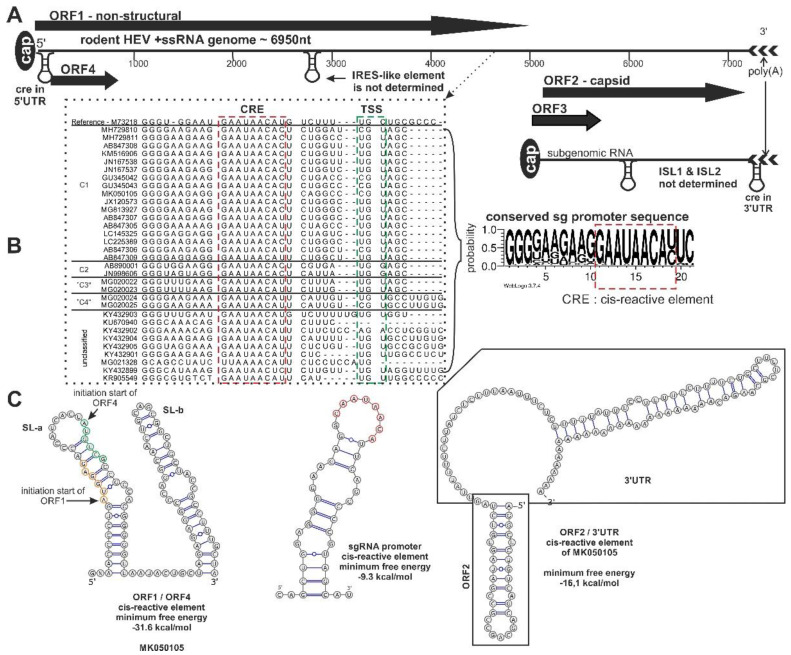
Schematic genome organization of rodent origin hepeviruses in species *Orthohepevirus C* and closely related, unassigned rodent hepeviruses. (**A**) The genome representation, (**B**) the significant nucleotide signals, and (**C**) the prediction of secondary RNA structural elements in rodent hepeviruses are presented. The alignment of the subgenomic RNA promoter sequence region (black dotted line), including the cis-reactive site (CRE) (red dotted line) and the transcription initiation site (TSS) (green dotted line), are highlighted. The consensus nucleotide Logo is generated from the alignment [49]. The internal ribosome entry site (IRES)-like, internal stem-loop (ISL)1/ISL2, CRE secondary RNA structural elements were predicted by ViennaRNA Web Service using the RNAfold server [50]. Open reading frame (ORF)4 is not present in all members of the orthohepevirus C.

The genome structure and organization of HEV in humans are studied [3,35,36,37,38,39,40,41], and rat hepeviruses are also summarized [3,34]. In the view of an increasing number of hepeviruses identified in rodents, this section aims to summarize the main features of the rodent hepevirus genome, and the main similarities/differences observed with human HEVs. The rodent hepevirus genome has a similar genome organization than human HEV. The average genome length of rodent hepeviruses are ~6950 nucleotides. The genome and subgenomic RNAs have an m^7^G-cap structure (7-methylguanosine) in the 5’ends and are polyadenylated (poly(A)) at the 3’end (Figure 1a) [37,38]. The subgenomic RNA (sgRNA) are generated from the negative-stranded RNA intermediate from the conserved subgenomic (sg) promoter nucleotide sequence [42]. The conserved site of the promoter acting as a cis-reactive element (CRE) and controls the synthesis of human HEV sgRNA with the help of the transcription start site (TSS) [42]. Both the CRE (GAAUGAAUAACAUGU, the CRE is underlined) and downstream, the TSS (UGC, conserved G is underlined) nucleotide motifs were present in rodent hepevirus sequences between the ORF1 and the ORF3/ORF2 putative initiation sites comparing to the reference HEV-1 (*Orthohepevirus A*) (Figure 1b) [42]. In HEV genome numerous secondary RNA structural elements (stem-loop) assist to the viral genome in replication and translation [37,43,44]. The most interesting structural element is the internal ribosome entry site (IRES) like element in the middle of the ORF1 region (Figure 1a) [44]. This signal similarly in picornaviruses (*Picornaviridae*) [45], or hepatitis C virus (*Flaviviridae*) [46] initiate the cap-independent translation of the novel ORF4 protein identified in species *Orthohepevirus A* [44]. Comparing the corresponding genomic region of rodent and human hepevirus sequences, only partial nucleotide sequence identity was found (data not shown), and the secondary RNA structure is undetermined in rodent hepeviruses. The other noteworthy SL-structure is the internal stem-loop structures (ISLs) in the central genomic region of the hepevirus capsid encoding region [43]. Both, ISLs (ISL1: CCGACAGAAUUAAUUUCGUCGG, ISL2: GUCGUCUCAGCCAAUGGCGAGCCGAC) RNA structures are highly conserved among species *Orthohepevirus A* and supposedly plays an important role in viral RNA synthesis, but, comparing this sequence with rodent hepeviruses, both motifs showed low sequence identity, especially in HEV-C1 species (*Orthohepevirus C*). Based on the sequence identity and the prediction of the secondary structure of rodent hepeviruses RNA, these ISL1 and ISL2 secondary RNA structures were not predictable. Oppositely, two stem-loop secondary RNA structures were identified in rodent hepeviruses similarly in reference species. One of them is located at the 5’end of the RNA genome forming two stem-loop (SL) structures (SLa and SLb) (Figure 1c). The SLa containing the initiation start codon of ORF1 and the putative ORF4 proteins, inducing the frame-shifting mechanism during translation, while the SLb could interact with ORF2 capsid protein and could play a role in viral encapsidation [47]. The other SL structure is located at the 3’end of the RNA genome (Figure 1c). This secondary RNA structure shows homology between the rodent and the reference HEVs [48]. In the reference strain Sar-55 (HEV-1), this is a critical element in virus replication, but in rodent hepeviruses the function of this element is still unclear.

**Figure 2:**
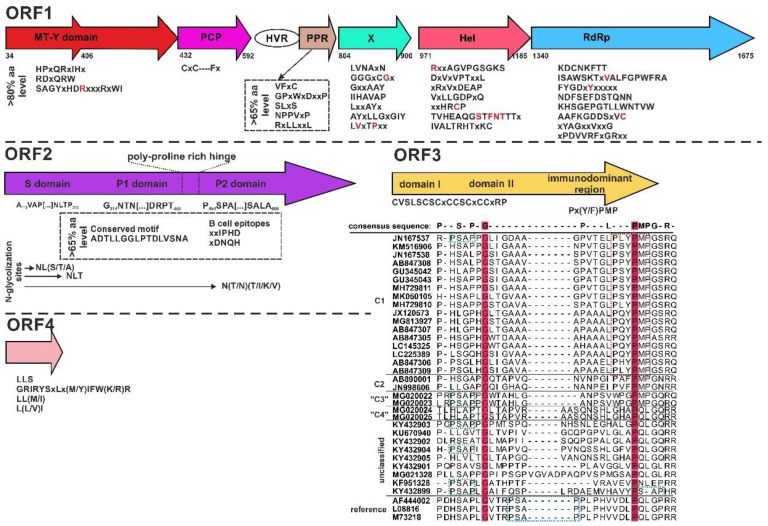
Schematic organization of the encoded polyproteins (ORF1, ORF2, and ORF3) and the putative (ORF4) protein in rodent origin hepevirus genome. The ORF1 is coding the non-structural proteins, sequentially the MT-Y: methyltransferase domain; PCP: papain-like cysteine protease domain; HVR: hypervariable region; PPR: poly-proline region; X: macro domain; Hel: helicase enzyme; RdRp: RNA-dependent RNA polymerase. The amino acid (aa) number positions are based on reference HEV-1 Burma strain. The conserved aa motifs in each ORFs were analyzed by GeneDoc using 65–80% aa conservation level set-up. The conserved aa is colored by black, the different aa is highlighted by red, and x means any aa in the motifs of rodent hepevirus ORF1. The S-, P1-, and P2 domains of ORF2-encoded viral capsid protein and the two main domains with the potential immunodominant region in ORF3-encoded protein are also identifiable in rodent hepeviruses. The immunodominant sites are emphasized by red, blue, and green dotted lines. The putative ORF4 in rodent hepevirus is not homologous with ORF4 of species *Orthohepevirus A*, but the conserved aa motifs are presented in the figure. The comparative analysis was based on the reference HEV-1 Burma strain (M73218), but other references, HEV-1 strain, Xinjiang (L08816), and strain Sar-55 (AF444002), were also involved in the analysis. Complete HEV-C1 strains used in this analysis: ratIDE079F (AB847305), ratELOMB-131 (LC145325), ratELOMB-187SF (AB847307), Vietnam-105 (JX120573), LCK-3110 (MG813927), ratESUMBAWA-140L (LC225389), ratESOLO-014SF (AB847306), ratIDE113F (AB847309), HEV 17/1683 (MK050105), rat/Mu09/0685/DEU/2010 (JN167537), R63 (GU345042), R68 (GU345043), rat/Mu09/0434/DEU/2010 (JN167538), LA-B350 (KM516906), ratESOLO-006SF (AB847308), GZ95 (MH729810), GZ481 (MH729811); HEV-C2: HEV-4351 (AB890001), FRHEV4 (JN998606), the putative HEV-“C3”: RdHEVAc14/LiJiang/2015 (MG020022), RdHEVAc86/LiJiang/2015 (MG020023), the putative HEV-“C4”: RdHEVEm40/LuXi/2014 (MG020024), RdHEVEm67/LuXi/2014 (MG020025) and novel unassigned hepeviruses: AlgSwe2012 (KF951328), Yunnan-2013 (KR905549), RtCm-HEV/XJ2016 (KY432903), RtCb-HEV/HeB2014 (KY432899), RtMg-HEV/XJ2016 (KY432902), RtEi-HEV/SX2016 (KY432904), RtCl-HEV/GZ2016 (KY432905), RtMr-HEV/HLJ2016 (KY432901), kestrel/MR22/2014/HUN (KU670940), 1 (MG021328). The partial capsid coding (ORF2) nucleotide sequence of strain NorwayRat/rat08-HEV/HUN/2018 (MT847624) was also involved into the analysis.

Four main polyproteins are translated from the open reading frames (ORFs) of HEV (Figure 2). The non-structural protein precursors from ORF1 [51], the viral capsid protein from ORF2 [52], the viroporin from ORF3 [53,54,55], and the putative regulatory protein from ORF4 [44], which were described in species *Orthohepevirus A*, and identifiable in rodent hepeviruses, too. These protein precursors and its significant amino acid (aa) motifs discussed below (Figure 2). The ORF1-encoded polyprotein contains non-structural proteins: methyltransferase domain (MT-Y), papain-like cysteine protease (PCP), hypervariable region (HVR), poly-proline region (PPR), macro (x) domain, helicase (Hel), and RNA-dependent RNA polymerase (RdRp) [37]. Based on the reference Burma strain, all conserved aa motifs described in HEVs so far present in rodent hepeviruses and a consensus sequence was calculated using strict consensus type in UGENE (threshold < 80%, http://ugene.net/) (Figure 2) [51,56]. The HVR and PPR are highly variable among rodent hepeviruses, too [36,51,57]. The function of HVR is a putative protein hinge. Absence of HVR may weaken the infectivity of the virus in vivo/vitro [58] and connecting with PPR, and both could be a key factor in the development of persistent HEV infections in immunocompromised patients [59]. The PPPAP and PPSAP aa motifs were described in the PPR [51]; however, these motifs were not identifiable in rodent hepevirus sequences. Comparing the sequences using a lower threshold <65%, VFxC, GPxWxDxxP, SLxS, NPPVxP, and RxLLxxL conserved aa motifs in ORF1 were found in rodent hepeviruses (Figure 2).

Towards the 3’end of the genome, two overlapping regions, the viral capsid protein encoded by ORF2 and a regulatory phosphoprotein with a multi-functional smaller protein called “viroporin” encoded by ORF3 is translated from the conserved sequence of the CRE of the sgRNA promoter sequence [42,60,61]. The ORF3 is overlapped with ORF2 and translated in a different reading frame. Comparing the HEV-C1 capsid protein sequences with the reference Burma strain, the shell (S) and the protruding (P) domains are separable in the N-terminal and C-terminal ends, respectively [52,61]. Further, the P domain is dissociated with P1 and P2 subdomains with a poly-proline rich hinge between the two-folds (Figure 2). Based on the strict consensus type in UGENE (threshold <65%) several conserved aa motifs are found in S and P domains of HEV-C1 (Figure 2). First of all, three N-glycosylation sites are predicted in the S and P2 domains (Figure 2). The NL(S/T/A) and the NLT motifs are hidden in the S domain, and the N(T/N)(T/I/K/V) motif is present in the P2 domain of the capsid protein. Secondly, the conserved ADTLLGGLPTDLVSNA aa motif was observed in the P1 domain. This site is a potential sugar-binding site in P1 domain, which is strictly conserved among species in *Orthohepevirus A* and this peptide plays an important role in binding and penetrating different cell lines susceptible to HEV [61,62]. Additionally, two conserved B-cell type epitopes, the xxIPHD and xDNQH aa motifs, were found in P2 domain, not only in species *Orthohepevirus A* and *B* but in rodent origin *Orthohepevirus C*, too [63].

The small ORF3-encoded protein is a cytoskeleton-associated phosphoprotein and seems to reduce the host inflammatory response and protect virus-infected cells during the pathway of transcription and translation [53,64]. The protein has two hydrophobic domains (domain I and II) in its N-terminus and two proline-rich regions in its C-terminus. Based on the reference Burma (HEV-1) sequence, the motif of the first proline-rich region is P/RMSPLR where the Ser-80 residue (underlined) is the main part of the motif. The other proline-rich region is PxxPxxP aa motif located in the central site of the ORF3 protein [54]. The PSAP aa motif in the late (L) domain and to date three different (P[TS]AP, PPXY, and YPDL) L domain motifs were studied. The function of this motif is to bind the host protein tumor suppressor gene 101 (Tsg101) subunit of the endosomal sorting complex required for transport (ESCRT) [55] and well-conserved in all mammalian HEVs (HEV-1 to HEV-4) in *Orthohepevirus A* and avian HEVs (gt1-gt3) in *Orthohepevirus B* [55,65,66]. However, several serine residues could be detected in rodent hepevirus sequences, but, based on the alignment, neither the first P/RMSPLR aa nor the second but aside from some exceptions, this motif is not PxxPxxP aa motifs present in rodent hepevirus sequences. Instead of that the CVSLSCSCxCCSCxCCxRP and the Px(Y/F)PMP aa (conserved proline residues underlined) motifs were found in species *Orthohepevirus C* (Figure 1) [67,68].

The modes of translation of ORF1, ORF2, and ORF3 proteins are cap-dependent [37], but the translation of the putative ORF4 protein identified in HEV-1 is dependent on an IRES-like element [44]. The RNA sequence positioning in the HVR and PPR region in ORF1 of HEV-1 viruses that forms the IRES-like element is not found in rodent hepevirus strains. However, low sequence identity was discovered in the putative gene region, open reading frame (a frame longer than 100bp) was not identified by the ORF Finder (https://www.ncbi.nlm.nih.gov/orffinder/) in that position. Among the significant putative ORFs described in silico analysis so far, the most prominent candidate is the protein located at the 5’ end of the genome overlapping with ORF1, but in a different frame (Figure 1) [14,31]. This putative, functionally active protein transcribed from ORF4 has some interesting properties. The approximately 183 aa-long protein-encoding ORF4 is marked by an abundance of leucine (mean 17.6%), arginine (mean 11.95%), and serine (mean 9.6%) residues, and these aa residues seem to be conserved among rodent origin hepeviruses [31]. In a later study, the putative ORF4 protein is experimentally detected and characterized by in vitro methods, but ORF4 has no known role in viral replication in rodent hepeviruses, and, to date, the in vivo function of this putative protein is still unclear [68]. To date, this protein was described from rodent origin hepeviruses, but it seems this protein only exists in hepeviruses identified from rat hosts, or hepeviruses which are genetically closely related to HEV-C1/C2 species. Hepeviruses (phylogenetically unassigned rodent hepeviruses and the tentative HEV-“C4” lineages) identified from murine hosts do not encode the ORF4 protein in their RNA genome [26]. Interestingly, bat hepeviruses are also encoded a 219-aa-long putative protein in their 5’end of the genomes. This protein contains 13.2% leucine, 6.4% arginine, and 11.4% serine residues and shows 16% aa identities in the corresponding aa region to rodent origin hepevirus sequences. Further, more in-depth comparative studies of the ORF4 proteins of human and rat hepeviruses could determine the exact role of the protein and the mechanism of transcription in the viral life cycle.

## 5. Phylogenetic Analysis of Rat Hepeviruses

**Figure 3:**
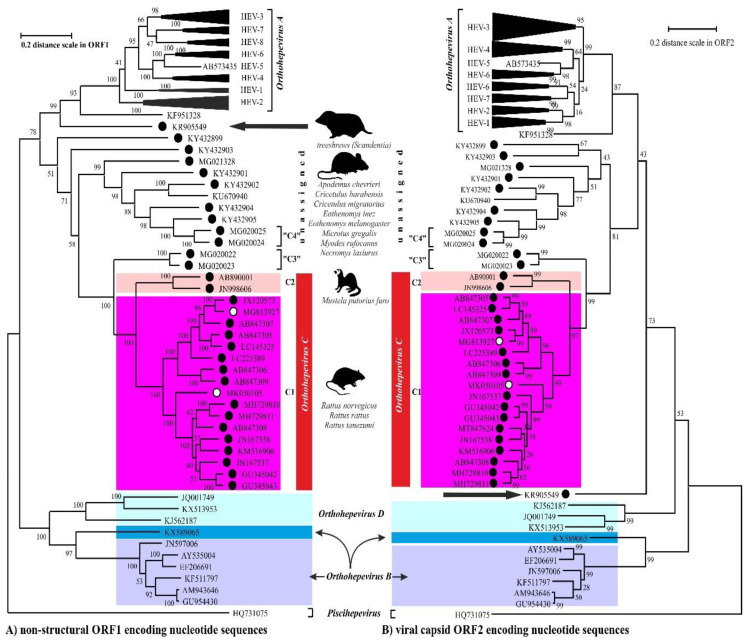
The phylogenetic reconstruction of hepeviruses. Phylogenetic trees are generated based on the (**A**) ORF1-encoded non-structural polyprotein (left) and (**B**) ORF2-encoded capsid protein (right) sequences and each tree are rooted to piscihepevirus A (*Piscihepevirus*) srain Heenan88 (HQ731075). Each phylogenetic tree is focusing on the analysis of the rodent origin HEV-C1 (magenta), HEV-C2 (pink) sequences in *Orthohepevirus C* (red) complementing with the currently unassigned rodent-borne hepevirus sequences. The putative HEV-“C3” and HEV-“C4” clades in *Orthohepevirus C* are also highlighted. The rodent hepeviruses are marked with black dot, while rodent origin HEV-C1 in human infections are highlighted by white dot. The reported (potential) source of infection of hepeviruses are shown next to the lineages. The phylogenetic subtrees are compressed based representative genotypes in *Orthohepevirus A.* The following sequences are used in subtree HEV-1: AY230202, MH918640, AY204877, X98292, L08816, D11092, LC225387, M73218, JF443721, FJ457024; HEV-2: MH809516, KX578717; HEV-3: FJ705359, KP294371, FJ998008, MF959764, LC260517, MF959765, KU513561, MK390971, JQ013794, JQ953664, AB301710, AP003430, KT633715, AF082843, AY115488, AB369689, AB369687, EU723512, EU360977, AB290313, AB248521, JQ026407, AF455784, FJ906895; HEV-4: DQ450072, AB108537, MK410048, AB369688, AB220974, AB074915, KU356182, KF736234, AB197673, AY723745, AJ272108, LC428039, DQ279091; HEV-5: AB573435; HEV-6: AB856243, AB602441; HEV-7: KJ496144, KJ496143; HEV-8: MH410176, KX387867, KX387865. The evolutionary analyses were conducted in MEGA X [71], and phylogenetic trees were inferred by using the Maximum Likelihood method and Jukes-Cantor model [72]. The tree is drawn to scale, with branch lengths measured in the number of substitutions per site.

**Figure 4:**
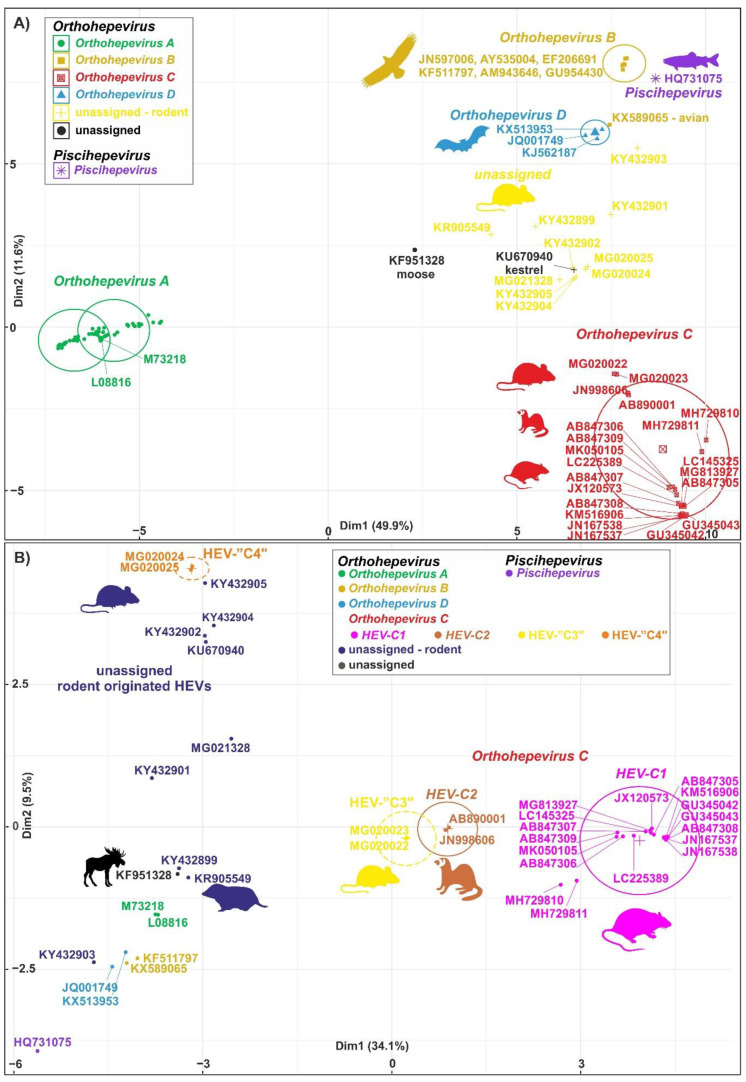
Cluster analysis of hepeviruses based on the complete nucleotide sequences of open reading Figure 1 (ORF1). (**A**) Plot A represents the distribution of representative hepevirus species in *Hepeviridae*, and (**B**) Plot B focuses on the distribution of rodent origin hepeviruses. *Piscihepevirus A* (purple), *Orthohepevirus A* (green)*, B* (gold)*, C* (red), and *D* (blue) and currently unassigned (yellow and black) species in Plot A and Plot B, respectively, are K-means clustered together [73]. The rodent origin hepevirus clusters in *Orthohepevirus C*, HEV-C1 (magenta), HEV-C2 (brown), and two novel clusters, HEV-“C3” (yellow) and HEV-“C4” (orange), are shown in Plot B. Briefly, a total of 101 and 40 complete coding sequence (CDS) of ORF1 sequences in Plot A and Plot B, respectively, are aligned by MEGA using codon based ClustalW alignment method and analyzed by R using seqinr, msa, cluster, factoextra, and randomColoR packages. An identity matrix of pairwise distances from aligned nucleotide sequences was calculated. The matrix scaled and the optimal number of clusters and the means (centres) of each cluster were estimated. The R algorithm code could be provided on request.

The members of the species *Orthohepevirus C* are constituted a distinct group. The nucleotide sequence p-distance within groups of species HEV-C1 and HEV-C2 seems to be low, from 4.2 ± 2.1% to 20.9 ± 6.1% throughout the nucleotide coding region (CDS) of ORF1-ORF4 in each group (data not shown). With a comparison, the mean p-distance scores are 9.7%, 28.9%, 19.7%, 17.4%, 23%, 15.1%, 9.7% within HEV-1, HEV-2, HEV-3, HEV-4, HEV-6, HEV-7, and HEV-8 groups in *Orthohepevirus A*, respectively. The cluster analysis is a common technique to classify or grouping data (sequences) to provide evolutionary connections or map the relationship between the related species [69,70]. The rodent hepeviruses form a discrete phylogenetic cluster among species *Orthohepevirus C* in family *Hepeviridae* (Figure 3). Based on the phylogenetic analysis (agglomerative, single linkage clustering) these sequences are separable at least four different clusters (C1, C2, “C3”, and “C4”), showing the evolutionary/phylogenetic position of one sequence in among similar/homologous species. Complementing the phylogenetic analysis with k-means cluster analysis, not only the distances between individual sequences can be observed more clearly, but intergroup relationships among rodent origin hepeviruses could be more perceptible (Figure 4) [69]. The analysis shows that HEV-C1, C2, and the tentative “C3” group of viruses in *Orthohepevirus C* could evolve together and may co-evolve with different rodent hosts. The viruses currently thought to be in the HEV-“C4” group are genetically distant from rat hepeviruses, but these viruses are closely related to the hepeviruses found in rodents in family Cricetidae based on the distance calculation between group mean distance: HEV-C1 to C2 is 43.8%, to “C3” is 50.8%, to “C4” is 67.7%, and to unassigned rodent origin hepeviruses is 69.4%, respectively (Figure 4b). Estimating of the average evolutionary divergence over sequence pairs within groups were 24.3% (standard error: 0.54%), 20.4% (s.e.: 0.68%), 8% (s.e.: 0.4%), 11.8% (s.e.: 0.5%), and 65.7% (s.e.: 1.08%) in HEV-C1, HEV-C2, HEV-“C3”, and HEV-“C4” viruses and unassigned rodent-origin (kestrel and common vole) hepeviruses by MEGA software. The mean diversity in the entire population is 51% (s.e.: 0.9%). It seems hepeviruses identified from rodents are genetically much more diverse than hepeviruses phylogenetically classified into cluster *Orthohepevirus A*. It is conceivable that rodents may be one of the key hosts and/or sources of the spread of hepevirus.

## 6. Animal and Cell Culture Models of Rat Hepevirus

The replication cycle and the cell receptor usage is not known for any of the hepeviruses but many host factors are involved in cell attachment and/or entry [74]. The initial in vivo investigations of the rat hepevirus as human pathogen was controversial. Immunocompetent and seronegative rhesus macaques do not appear to be susceptible to experimental infection with North American rat hepevirus strain, indicating that it is not a source of human infection [16]. Similarly, experimental infections of the domestic pig with rat HEV-C1 failed [75]. Rat hepevirus from human fecal sample shows abortive viral replication in A549 (human lung cancer cell line), Huh-7, and Caco-2 cells lines [12]. However, rat HEV-C1 from liver homogenate replicated efficiently in human hepatoma cell lines, PLC/PRF/5, HuH-7, and HepG2 but not in A549 in another study [76]. In light of the successful human infection and symptomatic clinical illness by rat hepevirus (see the next paragraph), the experimental results of rat hepevirus should be revisited.

## 7. Rat Hepevirus (HEV-C1) Infections in Human and Roles in Disease

The zoonotic potential of HEV-C was first suggested, as enzyme-linked immunosorbent assays (ELISAs) with *E. coli* expressed capsid protein indicated possible subclinical infection with HEV-C among forestry workers in Germany [77] and febrile inpatients in Vietnam [78]. The first confirmed human infection associated with rat hepevirus was detected from 1 (1.9%) of the 52 solid organ transplanted and immunocompromised patients with persistent hepatitis in Hong Kong, in 2017 [12] (Table 3). The 56-year-old man had abnormal liver function tests at day 59 post-liver transplantation and only the RT-PCRs using the pan-orthohepevirus primers and sequencing confirmed the presence of rat origin HEV-C1 in plasma, feces, saliva, and liver tissue specimens [12]. The patient was successfully treated with a 7-month-long oral ribavirin therapy. The source of the infection is potentially associated with rodent droppings in his living environment [12]. Based on this result, a 31-month-long prospective investigation found another 6 (0.27%) of 2201 patients with hepatitis and 1 (0.15%) of 659 immunocompromised persons with rat HEV-C1 infections including five patients in two neighbouring districts in Hong Kong between 2017 and 2019 [34] (Table 3). Clinically, the orthohepevirus C hepatitis was generally milder than orthohepevirus A hepatitis with lesser mean peak ALT (alanine-aminotransferase) and bilirubin [34]. Expanding the geographic distribution of severe hepatitis caused by rat HEV-C1 was reported from Canada as a potential imported infection from Africa [13]. On the other hand, rat HEV-C1 RNA was not detected by RT-PCR from sera collected from 162 patients with acute hepatitis tested positive by HEV IgM ELISA based on viruses in species *Orthohepevirus A* in Hungary [79]. These first reports support that viruses in species *Orthohepevirus C* are capable of causing human infection and disease, including acute and persistent hepatitis and subclinical infection. These results show that, maybe, immunocompromised patients seem to be more susceptible and at risk for these types of infection than immunocompetent patients. One of the most important questions is the frequency and the transmission mode of the infection because the exact transmission route of orthohepevirus C to human is not confirmed. Humans may have been infected directly via contact with environmental surfaces contaminated by rat droppings [12] or indirectly by contamination of water or food products (e.g., consumption of rat meat or adulterated meat products). The transmission of orthohepevirus C from rat (or rodents) through the intermediate animal host (e.g., domestic animals, etc.) to humans is also an open question, as well as the role as blood-borne pathogen through blood transfusion and/or organ transplantation. Mapping the transmission routes of orthohepevirus C are necessary to prevent human infections. Ribavirin could be an effective treatment against HEV-C1 [12].

## 8. Laboratory Detection of Rat Hepevirus (HEV-C1) in Human

The presently used human HEV diagnostics are designed based on the HEV-1 to HEV-4 in *Orthohepevirus A.* No serologic methods are commercially available for detection of antibodies against HEV-C1 or other orthohepevirus C viruses in human. Potential cross-reacting antibodies were recognized between the HEV-C1 and orthohepevirus A antigens using IgM/IgG ELISA kits and Western blot methods [12,18,34]; however, this connection is uncertain, unexplored, and not validated. Recent studies reported that preliminary HEV-A antibodies do not protect against HEV-C infection, and vice versa [12,80]. On the other hand, the use of truncated HEV-3 and HEV-C1 capsid protein derivatives allowed the differentiation of antibody responses [77]. According to current knowledge, the laboratory diagnosis cannot be based on serology method because of the risk of false negativity. The most reliable method to determine orthohepevirus C infection is to detect viral genomic RNA by RT-PCR [14,15], including nested broad-spectrum RT-PCR [15]. However, designing and selecting specific primer sequences is the crucial point of the sensitivity of this method. The commonly used primers for human HEV (which are designed based on orthohepevirus A viruses) are unable to detect the HEV-C genome in patient’s specimens [12]. It should be also noted that at present the genetic diversity of HEV-C1 strains in rat (or potentially in other animals) are currently not known, and it has an effect on the sensitivity of the molecular methods, too. HEV-C1 was detected by RT-PCR using specific primers in serum, feces, saliva, and liver tissue in human infection, and feces contained the highest RNA load [12].

## 9. Summary and Future Directions

The history of the science of HEVs continues to abound in surprise. It ran a great career from a neglected tropical acute waterborne disease, then a zoonotic infection from animals to humans in developed countries through as an etiological agent in chronic hepatitis and extrahepatic diseases in human. Now, we should re-evaluate the knowledge of hepevirus again. Evidence shows that the animal reservoir of the human hepevirus infections is proven to have been supplemented with rats, and, not only orthohepevirus A, but the member of the orthohepevirus C viruses are also able to cause human zoonosis among both immunocompromised and immunocompetent patients with hepatitis. This novel knowledge has a consequence to the epidemiology, clinical aspects, laboratory diagnosis and prevention of HEV infection in human.

Opening of this novel chapter could not answer yet for several important questions. Further studies needed to investigate how frequent is the HEV-C1 infection in human and what percentage of human HEV disease misdiagnosed because of this hepevirus variant. In addition to this, it should explore the wideness of genetic (and antigen) diversity of human pathogen HEV-C1 in rat, rodents and potential in other animal hosts. It should be studied that are there any differences in zoonotic potential between different rat hepeviruses to human. One of the central issues is to localize the exact hot-spots, the transmission mode(s) from animal to human, and geographic distribution of HEV-C infections. All potential transmission modes should be investigated including the organ and blood transfusion risks in a certain geographic area especially in the situation if the donor is infected asymptomatically. As in orthohepevirus A viruses the full disease spectrum (acute and chronic hepatitis and extrahepatic diseases) of HEV-C1 infections should be investigated in humans. Clinicians are aware that rats are a potential reservoir of human hepevirus infections, too.

HEV-C1 (species *Orthohepevirus C*) is genetically and antigenically different from viruses in species *Orthohepevirus A*. At the same time, the presently used human (serological and molecular) diagnostics are designed based on certain genotypes (most of the HEV-1 and HEV-3) of orthohepevirus A viruses. These means that these methods have low sensitivity for HEV-C1 [12,80]. HEV-C1 infections presently undiagnosed because of i) low antigen cross-immunity in serological test and ii) amplification failure in RT-PCRs because of primer mismatch [12]. Serologic tests should be developed which can detect both orthohepevirus A and orthohepevirus C antibodies (at the same time) in humans by ELISA, and we need to extend the coverage of the diagnostic primers and probes for both HEV-A and HEV-C (particularly HEV-C1) in molecular methods.

Confirmation of HEV-C1 infections and symptomatic diseases in humans re-opened the long-lasting and full of surprises story of HEV in human. Rat HEV-C1 is also proving that further studies are needed to explore the full animal reservoir, cross-species transmission, and genetic diversity of hepeviruses potentially threatening humans.

## Figures and Tables

**Table 1:** Summary of known hosts, potential animal reservoirs, and known sources of orthohepeviruses for human.

	Genus	Species	Animal Host or Reservoir *
*Hepeviridae*	*Orthohepevirus*	*Orthohepevirus A*	**human**, **swine**, **wild boar**, **deer**, **rabbit**, **camel**, goat, bottlenose dolphin, cattle, sheep, mongoose, yak, rat, nonhuman primates
		*Orthohepevirus B*	chicken
		*Orthohepevirus C*	**rat**, shrew, voles, brown bear, ferret, mink kestrel/falcon, red fox
		*Orthohepevirus D*	bats
		unassigned orthohepeviruses	moose, little egret
	*Piscihepevirus*	*Piscihepevirus A*	cutthroat trout (fish)

* Bold indicates animals in which zoonotic infection has been confirmed.

**Table 2:** Summary of rat hepevirus (hepatitis E virus (HEV)-C1) molecular studies in the literature.

Order/Family	Species	Methods, Sample Type and Positivity Rate	Collection Date and Place	HEV Type	GenBank Acc. No	Reference
Rodentia/Muridae	Norway rat (*Rattus norvegicus*)	2 RT-PCR+/30 fecal samples	spring of 2007 and 2008 (Hamburg; Germany)	HEV-C1	GQ504009, GQ504010	[14]
	Norway rat (*Rattus norvegicus*)	2 RT-PCR+/6 liver samples	July 2009 (Hamburg; Germany)	HEV-C1	GU345042, GU345043	[15]
	Norway rat (*Rattus norvegicus*)	2 HEV IgM+/134 sera; 1 RT-PCR+/2 HEV IgM+ sera	2003 (Los Angeles; USA)	HEV-C1	JF516246	[16]
	Norway rat (*Rattus norvegicus*) Tanezumi rat (*Rattus tanezumi*)	5 HEV IgM+/139 sera; 1 RT-PCR+/5 HEV IgM+	2011 (Haiphong and Hanoi; Vietnam)	HEV-C1	JN040433 (=JX120573)	[18]
	Norway rat (*Rattus norvegicus*) and Black rat (*Rattus rattus*)	35 RT-PCR+/446 liver samples	<2012 (15 states; USA)	HEV-3 (*N* = 34) and HEV-C1 (*N* = 1)	JQ898480–JQ898514	[29]
	Norway rat (*Rattus norvegicus*)	14 RT-PCR+/101 liver samples	2008-2010 (Hamburg, Berlin, Stuttgart, Esslingen; Germany)	HEV-C1	JN167530–JN167538	[30]
	Norway rat (*Rattus norvegicus*), Greater bandicoot rat (*Bandicota indica*), *Rattus flavipectus*, *Rattus rattoides losea*	59 HEV IgM+/713 sera; 12 RT-PCR+/59 HEV IgM+	Dec 2011-Sept 2012 (Zhanjiang, China)	HEV-C1	KC465998–KC465999	[19]
	Black rat (*Rattus rattus*)	17 RT-PCR+/116 sera	Aug 2011-Febr 2012 (Lambok Island; Indonesia)	HEV-C1	AB725884–AB725900	[17]
	Black rat (*Rattus rattus*)	99 RT-PCR+/369 sera	Sept-Oct, 2012 (Solo, Mataram, Indonesia)	HEV-C1	AB847305–AB847406	[31]
	Norway rat (*Rattus norvegicus*) and Black rat (*Rattus rattus*)	63 RT-PCR+/508 liver samples	2005–2016 (12 European countries)	HEV-C1 (*N* = 49) HEV-3 (*N* = 1)	KX774641–KX774673, KY938011–KY938027	[20]
	Black (*Rattus rattus)* and Norway rats (*Rattus norvegicus)*	9 RT-qPCR +/109 liver and chest cavity fluid samples	2014-2017 Lithuania	HEV-C1	MH400712–MH400717	[21]
	Black rat (*Rattus rattus*)	2 RT-PCR+/242 sera	2014-2016 (Bali and Sumbawa, Indonesia)	HEV-C1	LC225388–LC225389	[32]
	Norway rat (*Rattus norvegicus*)	8 RT-PCR+/61 liver samples	2014-2016 (Great Britain)	HEV-C1	MK770165–MK770171	[33]
	Norway rat (*Rattus norvegicus*)	7 RT-PCR+/159 rectal swabs	2018-2019 (Hong-Kong, China)	HEV-C1	MN450855, MN450859–MN450864	[34]
	Norway rat (*Rattus norvegicus*)	1 RT-PCR+/10 fecal samples	2017-2018 (Budapest, Hajdúböszörmény; Hungary)	HEV-C1	MT847624	in this study
Soricomorpha/Soricidae	Asian musk shrew (*Suncus murinus*)	12 HEV IgM+/260 sera; 5 RT-PCR+/12 HEV IgM+ sera	December 2011-September 2012 (Zhanjiang City; China)	HEV-C1	KC465990–KC466001	[22]

**Table 3:** Summary of human infections (*N* = 9) associated with rat origin HEV-C1 virus (species *Orthohepevirus C*) in the literature. HBV = hepatitis B virus.

Patient	Underlying Disease	Acute Disease	Potential Source of the Infection	Geographic Region (Year)	GenBank Acc. No	Reference
56-year-old man	liver transplantation, HBV carrier, immunocompromised	persistent hepatitis	rodent droppings	Hong Kong (2017)	MG813927	[12]
71-year-old female	rheumatoid arthritis, immunocompromised	acute hepatitis	unknown	Hong Kong (2017)	MN450851	[34]
67-year-old man	kidney transplantation, immunocompromised	persistent hepatitis	unknown	Hong Kong (2018)	MN450852	[34]
74-year-old man	kidney transplantation, HBV carrier, immunocompromised	persistent hepatitis	unknown	Hong Kong (2018)	MN450853	[34]
81-year-old man	prostate cancer	acute hepatitis	unknown	Hong Kong (2019)	MN450856	[34]
73-year-old man	none, immunocompetent	acute hepatitis	unknown	Hong Kong (2019)	MN450857	[34]
67-year-old man	metastatic cancer	subclinical	unknown	Hong Kong (?)	MN450858	[34]
43-year-old man	HIV infection	persistent hepatitis	unknown	Hong Kong (2019)	MN450854	[34]
49-year-old man	none, immunocompetent	severe acute hepatitis	unknown	Canada(may be imported from Uganda, Africa) (2019)	MK050105	[13]
